# Cu_2_O/CuO Bilayered Composite as a High-Efficiency Photocathode for Photoelectrochemical Hydrogen Evolution Reaction

**DOI:** 10.1038/srep35158

**Published:** 2016-10-17

**Authors:** Yang Yang, Di Xu, Qingyong Wu, Peng Diao

**Affiliations:** 1 Key Laboratory of Aerospace Materials and Performance (Ministry of Education), School of Materials Science and Engineering, Beihang University, Beijing 100191, P. R. China

## Abstract

Solar powered hydrogen evolution reaction (HER) is one of the key reactions in solar-to-chemical energy conversion. It is desirable to develop photocathodic materials that exhibit high activity toward photoelectrochemical (PEC) HER at more positive potentials because a higher potential means a lower overpotential for HER. In this work, the Cu_2_O/CuO bilayered composites were prepared by a facile method that involved an electrodeposition and a subsequent thermal oxidation. The resulting Cu_2_O/CuO bilayered composites exhibited a surprisingly high activity and good stability toward PEC HER, expecially at high potentials in alkaline solution. The photocurrent density for HER was 3.15 mA·cm^−2^ at the potential of 0.40 V *vs*. RHE, which was one of the two highest reported at the same potential on copper-oxide-based photocathode. The high photoactivity of the bilayered composite was ascribed to the following three advantages of the Cu_2_O/CuO heterojunction: (1) the broadened light absorption band that made more efficient use of solar energy, (2) the large space-charge-region potential that enabled a high efficiency for electron-hole separation, and (3) the high majority carrier density that ensured a faster charge transportation rate. This work reveals the potential of the Cu_2_O/CuO bilayered composite as a promising photocathodic material for solar water splitting.

Harvesting sunlight to produce clean chemical fuel offers a desirable and sustainable approach toward fulfilling the need for clean energy with minimal environmental impact^1^,^2^. Among many photo-to-chemical energy conversion processes, hydrogen evolution from solar water splitting is one of the most promising approaches^3^,^4^,^5^,^6^ because the produced H_2_ can serve directly as a fuel without producing pollutants or green-house gases upon combustion. Solar water splitting involves two half-cell reactions: the hydrogen evolution reaction (HER) and the oxygen evolution reaction (OER), and the kinetics of which are quite different^3^,^7^. To achieve efficient water splitting, it is necessary to separately investigate each half-cell reaction. However, in most photocatalytic water splitting systems, HER and OER usually occur at different regions of one tiny catalyst particle^3^,^7^, making it impossible to spatially separate the two half-cell reactions and then difficult to individually study each of them.

Photoelectrochemical (PEC) cells are widely used as solar water splitting devices^8^,^9^,^10^, not only because they combine solar energy collection with water electrolysis, but also because they spatially separate HER and OER, and thus making it possible to investigate the kinetics of each half-cell reaction. For a PEC water splitting process, the light energy is absorbed by semiconductor material to generate electron-hole pairs^3^,^7^,^8^,^9^,^10^, and the photoinduced electrons (or holes) are driven, by the space-charge field, to the semiconductor/solution interface where they reduce (or oxidize) water. Usually, n-type semiconductors, such as TiO_2_^8^, ZnO^11^, WO_3_^12^,^13^,^14^, Fe_2_O_3_^15^,^16^, CuWO_4_^17^, BiVO_4_^18^, and Ag_3_PO_4_^19^, whose valence band edges are more positive than the potential of the H_2_O/O_2_ redox couple, can be used as photoanodes for OER. While, p-type semiconductors with conduction band edge located more negative than the potential of H_2_O/H_2_ can be used as photocathode for HER^6^,^7^. For example, p-GaInP_2_^20^,^21^, p-InP^22^, p-WSe^23^, p-Si^24^,^25^,^26^, p-CdS^27^, and p-type copper oxides^28^,^29^,^30^,^31^,^32^,^33^,^34^,^35^,^36^,^37^,^38^,^39^,^40^,^41^,^42^,^43^,^44^,^45^,^46^,^47^,^48^,^49^,^50^,^51^,^52^ have been explored as possible candidates for water splitting photocathodes. Among these p-type semiconductors, cuprous and cupric oxides (Cu_2_O and CuO) are attractive due to their wide absorption band in visible region, high abundance in earth crust, ease of preparation, low cost and low toxicity to the environment.

Cu_2_O and CuO have a direct bandgap of approximately 2.0–2.5 eV^28^,^29^,^30^,^32^,^39^,^53^,^54^,^55^,^56^,^57^,^58^,^59^,^60^ and 1.3–1.7 eV^31^,^36^,^37^,^60^,^61^,^62^, respectively, depending on the preparation methods and conditions. The small bandgap energies allow Cu_2_O and CuO to absorb vast majority of solar spectrum, and the direct bandgaps endow the two oxides with a large absorption coefficient. Both Cu_2_O and CuO have been demonstrated to be active for photocatalytic water reduction^28^,^29^,^30^,^31^,^32^,^33^,^34^,^35^,^36^,^37^,^38^,^39^,^40^,^41^,^42^,^43^,^44^,^45^,^46^,^47^,^48^,^49^,^50^,^51^,^52^. However, the high electron-hole recombination rate in Cu_2_O and CuO prevent these two materials from being highly efficient photocathodes for HER. To address this issue, one strategy is to construct heterojunction structures with other semiconductors^5^, such as CuO/ZnO^63^,^64^, CuO/TiO_2_^65^, and Cu_2_O/TiO_2_^66^, for efficient separation of photogenerated electron-hole pairs. Since both the conduction and valence band edges of Cu_2_O are at more negative potentials than those of CuO^49^,^50^,^67^,^68^, the photogenerated electrons in the conduction band of Cu_2_O will be injected into that of CuO at the Cu_2_O/CuO interface under visible-light irradiation, whereas the photogenerated holes in the valence band of CuO are injected into that of Cu_2_O. As a result, the Cu_2_O/CuO heterojunction facilitates electron-hole separation and can improve photo-to-chemical energy conversion efficiency^41^,^42^,^47^,^48^,^49^,^50^,^51^,^67^,^68^.

It is desirable for a photocathodic material, especially copper-oxide-based materials, to be highly active at more positive potential region (>0.40 V vs. RHE) because of the following two reasons. (1) A more positive HER potential implies a lower overpotential for HER, and then a smaller external electrical energy input. (2) Cu_2_O and CuO are more stable at a higher potential for PEC HER because a high potential restrains the photoreduction of Cu_2_O (or CuO) to metal copper, which was believed to be the main cause of the low stability of copper oxides during PEC HER^28^,^31^. Unfortunately, nearly all of reported copper-oxide-based photocathodes showed very low activity toward PEC HER at high potential region^28^,^29^,^30^,^31^,^32^,^33^,^35^,^36^,^37^,^38^,^39^,^40^,^41^,^42^,^43^,^44^,^45^,^46^,^47^,^48^,^49^,^50^,^51^,^52^ though some of them exhibited high activity at low potential region^28^,^40^,^41^,^42^,^43^. Therefore, it is still a great challenge to develop copper-oxide-based photocathodes that are highly active toward HER at a more positive potential region.

In this work, we report a facile method that involved a repeated double-potential pulse chronoamperometric (r-DPPC) deposition and a subsequent thermal oxidation to prepare the Cu_2_O/CuO composite photocathodes. The resulting Cu_2_O/CuO bilayered composite exhibits surprisingly high PEC activity toward HER, especially at high potentials in alkaline solution. The photocurrent density obtained on the composite photocathode is 3.15 mA·cm^−2^ at 0.40 V *vs*. RHE, which is one of the two highest values reported on copper-oxide-based photocathodes at the same potential. This work not only shows the potential of the Cu_2_O/CuO bilayered composite as a photocathodic material for efficient HER, but also provides an ease, low-cost, and scalable strategy to prepare the Cu_2_O/CuO composite for hydrogen production.

## Results

### Morphology, structure and light absorption properties of copper oxides

**Figure 1:**
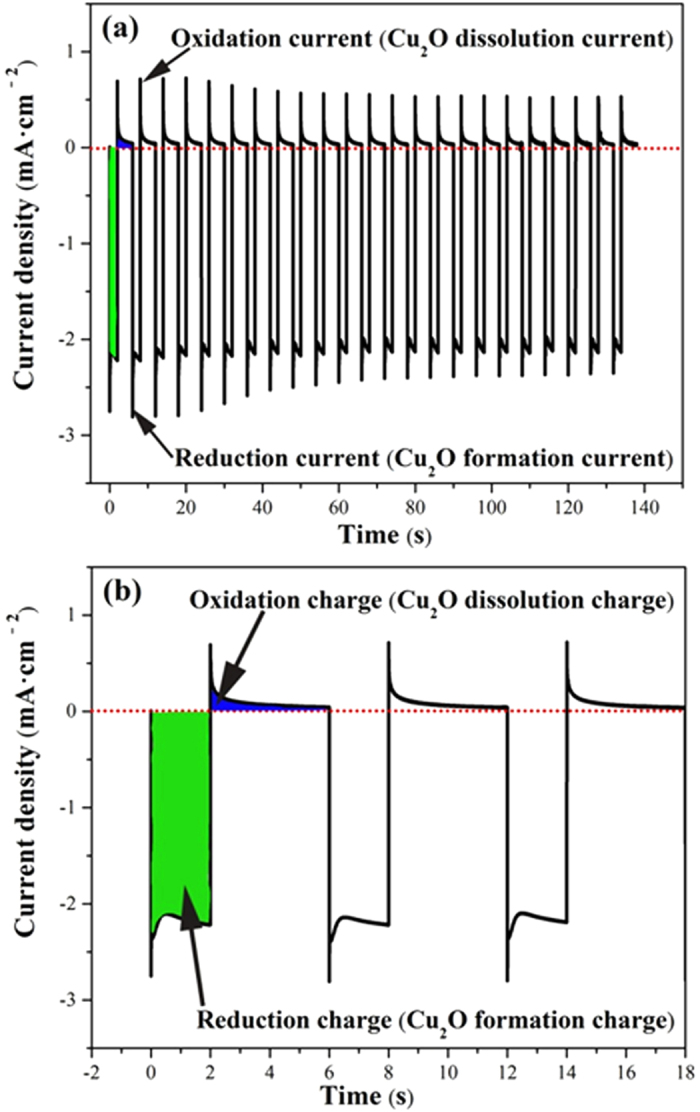
(**a**) Variation of current density as a function of time during repeated double-potential pulse chronoamperometric (r-DPPC) deposition in a solution containing 0.48 M CuSO_4_ and 3 M actic acid, which was adjusted to pH 9.2 with 5 M NaOH. (**b**) Enlarged first three cycles showing the negative charge used to grow Cu_2_O (the green shaded area) and the positive charge used to dissolve Cu_2_O (the blue shaded area) during one deposition-dissolution cycle.

We developed a “repeated deposition-dissolution” strategy to prepare Cu_2_O film on FTO substrates. This strategy was achieved by a repeated double-potential pulse chronoamperometric (r-DPPC) method that involved two potential pulses at −0.50 V and 0.0 V *vs.* SCE for 2 s and 4 s, respectively. Figure 1a shows the variation of current density as a function of time during r-DPPC deposition. The two opposite polarity currents observed in Fig. 1a indicates that both a reduction and an oxidation process occur in one DPPC deposition cycle. As we know that the equilibrium potential of the redox couple Cu^2+^/Cu_2_O in the deposition solution is ca. −0.06 V *vs.* SCE. The more negative but shorter pulse (at −0.50 V *vs.* SCE for 2 s) ensured a high reduction overpotential to grow Cu_2_O nanocrystals, while the subsequent more positive and longer pulse (at 0.0 V *vs.* SCE for 4 s) provided a mild oxidation overpotential to dissolve part of the Cu_2_O that was deposited on high energy sites during the preceding deposition process. Figure 1b shows the current response of electrode in the first three cycles of r-DPPC deposition, from which the negative charge used to grow Cu_2_O and the positive charge used to dissolve Cu_2_O in each deposition-dissolution cycle can be obtained by integrating the corresponding current-time curves. The integrated negative charge (the green shaded area) is ca. 4.4 × 10^−3 ^C·cm^−2^·cycle^−1^, which is much larger than the integrated positive charge (the blue shaded area, ca. 3.2 × 10^−4 ^C·cm^−2^·cycle^−1^), indicating that one deposition-dissolution cycle resulted in a net growth of the Cu_2_O. The one-cycle growth rate, which was obtained from the difference between the negative and the positive charges, is ca. 0.061 μg·cm^−2^·cycle^−1^. This value corresponds to a Cu_2_O growth thickness of ca. 10 nm per cycle (*ca*. 100 nm·min^−1^) when the density of Cu_2_O is taken as 6.0 g·cm^−3^ 69. The Cu_2_O films with certain thickness were obtained by repeating DPPC deposition for required time. The deposition-dissolution strategy greatly lower the net growth rate and improve the uniformity of the Cu_2_O film, as can be seen from the following SEM results.

**Figure 2:**
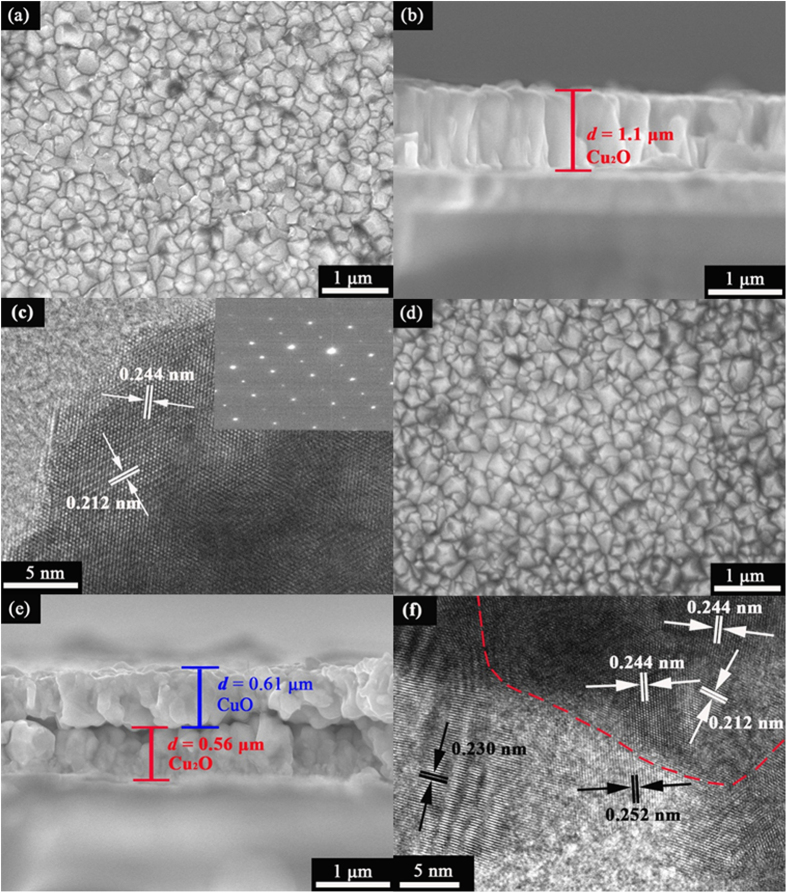
(**a**) Top-view, (**b**) cross-section-view SEM images and (**c**) HRTEM image and SAED pattern (inset) of Cu_2_O prepared by r-DPPCD for 10 min. (**d**) Top-view, (**e**) cross-section-view SEM images and (**f**) HRTEM image of the Cu_2_O/CuO bilayered composite prepared by thermal oxidation of Cu_2_O film in air at 400 °C for 2 h.

Figure 2a shows a typical top-view SEM image of Cu_2_O film prepared by r-DPPC deposition for 10 min (100 deposition-dissolution cycles) on an FTO substrate. The Cu_2_O film is composed of closely packed Cu_2_O nanocrystals that have an average grain size of ca. 265 ± 60 nm. The average grain size of Cu_2_O could be easily controlled in the range from 50 nm to 300 nm because it increased monotonically with increasing deposition time, as shown in Figure S1 in supplementary material. It should be pointed out here that, compared to the traditional constant potential deposition, r-DPPC deposition results in a much smaller grain size of Cu_2_O and a much narrower size distribution within the same deposition time. For example, as shown in Figure S2 of supplementary material, the Cu_2_O film prepared by r-DPPC deposition for 2.5 min had an average grain size of ca. 100 ± 25 nm, while the film prepared by constant potential deposition for the same deposition time had a much larger grain size of ca. 215 ± 95 nm. All these results provide solid evidence that r-DPPC deposition is superior to constant potential deposition in preparing more uniform and compact Cu_2_O films.

**Figure 3:**
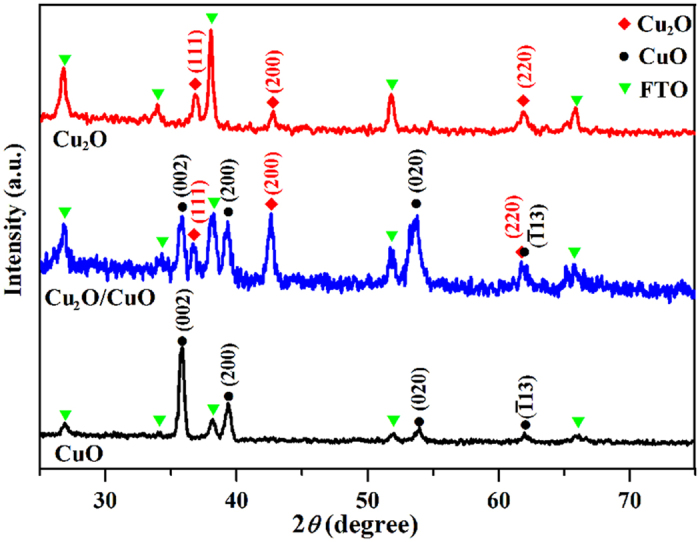
XRD spectra of the Cu_2_O, CuO, and Cu_2_O/CuO bilayered composite films prepared on FTO substrate. The Cu_2_O/CuO and CuO films were prepared by thermal oxidation of the FTO-supported Cu_2_O in air at 400 °C for 2h and 4 h, respectively.

Figure 2b shows a typical cross-section-view SEM image of Cu_2_O film prepared by r-DPPC deposition for 10 min (100 deposition-dissolution cycles). The deposited Cu_2_O film is a continuous layer with a thickness of about 1.10 μm, in good agreement to the value of 1.0 μm estimated from electrodeposition rate (ca. 100 nm·min^−1^). The thickness of the film can be controlled from ca. 0.09 nm to 1.10 μm by varying total deposition time from 1 min to 10 min (see Figure S3 in supplementary material). Figure 2c shows a typical HRTEM image and the SAED pattern of an individual Cu_2_O grain, which clearly indicate that the Cu_2_O grain has a single crystalline structure. The well-resolved lattice spacings in Fig. 2c are 0.244 nm and 0.212 nm, which can be assigned to the (111) and (200) planes of cubic Cu_2_O, respectively. The SAED pattern matches well with that of cubic Cu_2_O, in accordance with HRTEM results. XRD measurements were also carried out to analyze the crystal structure of the Cu_2_O film and the result was shown in Fig. 3. The XRD pattern of the Cu_2_O film agrees well with that of cubic Cu_2_O (JCPDS NO. 03-0898), further confirming that the film was composed of cubic Cu_2_O nanocrystals.

The Cu_2_O/CuO bilayered composite was fabricated by thermal oxidation of Cu_2_O film in air at 400 °C for 2 h^70^. As shown in Fig. 2d, the top-view morphology of the sample changes little after thermal oxidation. However, the cross-section SEM image (Fig. 2e) clearly indicates that the sample changes from a continuous film before thermal oxidation to a bilayer-structured film after thermal oxidation. This observation implies that the outer layer of the Cu_2_O film was transformed into CuO, and a Cu_2_O/CuO heterojunction was formed during thermal oxidation. Figure 2f shows the HRTEM image of the Cu_2_O/CuO composite. Besides the lattice spacings of cubic Cu_2_O (0.244 nm for (111) plane and 0.212 nm for (200) plane), the lattice spacings of 0.252 nm and 0.230 nm are also observed, which correspond to the (002) and (200) planes of monoclinic CuO, respectively. HRTEM provide solid evidence that there exists a Cu_2_O/CuO heterojunction in the composite, as shown in the dashed line in Fig. 2f. The XRD pattern of the Cu_2_O/CuO bilayered composite is shown in Fig. 3 (the blue line), in which the diffraction peaks of both cubic Cu_2_O and monoclinic CuO appear, providing direct evidence for the formation of the Cu_2_O/CuO composite.

The thickness of the Cu_2_O and the CuO layers in Cu_2_O/CuO composite varied with the thermal oxidation time. Typical cross-section SEM images of the Cu_2_O/CuO bilayered composite prepared with different thermal oxidation time are shown in Figure S4 of supplementary material. As the thermal oxidation time was increased from 0.5 h to 2 h, the thickness of the outer CuO layer increased from ca. 0.09 μm to ca. 0.61 μm, while the thickness of the inner Cu_2_O layer decreased from ca. 1.08 μm to ca. 0.56 μm. In addition, after thermal oxidation in air at 400 °C for 4 h, the bilayered-structure disappeared and a continuous film was observed again, indicating that the entire Cu_2_O layer has been oxidized to CuO. XRD pattern (black) in Fig. 3 confirms that the cubic Cu_2_O film was completely transformed into monoclinic CuO film after thermal oxidation for 4 h. On the basis of cross-section SEM images, the variation of layer thickness of Cu_2_O and CuO as a function of thermal oxidation time was obtained, as shown in Figure S4d of supplementary material. All these results provide direct evidence that we could easily control the Cu_2_O-to-CuO thickness ratio by varying the thermal oxidation time.

**Figure 4:**
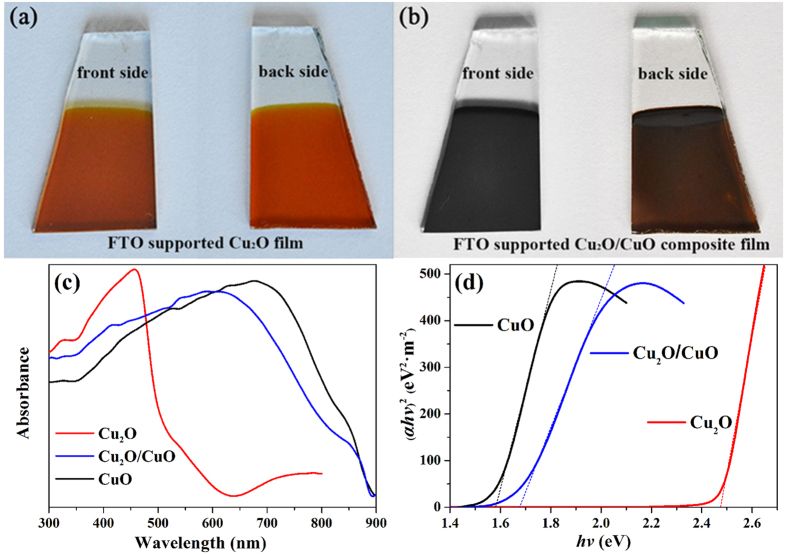
The optical images of (**a**) FTO-supported Cu_2_O film and (**b**) FTO-supported Cu_2_O/CuO bilayered composite film. (**c**) UV–vis diffuse reflectance spectra of the pure Cu_2_O (read line), pure CuO (black line), and Cu_2_O/CuO (blue line) composite films prepared on FTO substrates. (**d**) Tauc plots of Cu_2_O, CuO, and Cu_2_O/CuO composite films.

The light-absorption properties of the Cu_2_O and the Cu_2_O/CuO composite films are very important when they are used as photocatalysts for PEC HER. Figure 4a,b show the optical images of an FTO-supported Cu_2_O film before and after thermal oxidation in air at 400 °C for 2 h, respectively. The Cu_2_O film prepared by r-DPPC deposition exhibits a yellowish-red color (Fig. 4a)^71^, however, the front side of the film changes its color to black after thermal oxidation (Fig. 4b), indicating the formation of CuO layer^39^. The yellowish-red color can still be distinguished from back side of the film (Fig. 4b), implying that the inner layer of Cu_2_O survived the thermal oxidation. Therefore, we can safely conclude that after thermal oxidation, the film has a bilayer structure that is composed of an inner Cu_2_O layer and an outer CuO layer, and there must be a Cu_2_O/CuO heterojunction between the two layers.

Figure 4c shows the UV-Vis diffuse reflectance spectra of the Cu_2_O, CuO, and Cu_2_O/CuO composite films. The pure Cu_2_O have an absorption edge at ca. 600 nm, while the Cu_2_O/CuO bilayered composite extends the absorption edge to ca. 900 nm due to the low band gap energy of CuO. Tauc plot, which were obtained from UV-Vis spectra, is usually used to determine the band gap energy of semiconductor based on the following equations:^72^


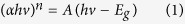


where *α* is the absorption coefficient that can be obtained from UV-Vis spectra, *hv* is the energy of photon, *A* is a constant, *E*_g_ is the optical band gap energy, and *n* depends on the nature of the transition. For direct transition n is 2 and for indirect transition n is 1/2. As shown in Fig. 4d, straight lines are obtained when (*αhv*)^2^ is plotted against photon energy (*hv*), indicating that the absorption is due to a direct transition for both Cu_2_O^53^,^54^,^55^,^56^,^57^,^58^,^60^ and CuO^36^,^37^,^60^,^61^,^62^. The band gap energies of Cu_2_O and CuO, which can be obtained from the intercept on the abscissa, are 2.47 eV and 1.58 eV, respectively. These values are in good agreement with those previous reported for Cu_2_O^54^,^58^,^59^ and CuO^31^,^37^,^60^. Figure 4d also shows that the apparent band gap energy of the Cu_2_O/CuO bilayered composite is 1.67 eV, suggesting that the combination of Cu_2_O and CuO extends the light absorption range. As the Cu_2_O/CuO composite is used as photocathode material for PEC HER, the extension of light absorption range may significantly improve the photo-to-hydrogen conversion efficiency.

### Activity and stability of Cu_2_O/CuO composite photocathodes for PEC water splitting

**Figure 5:**
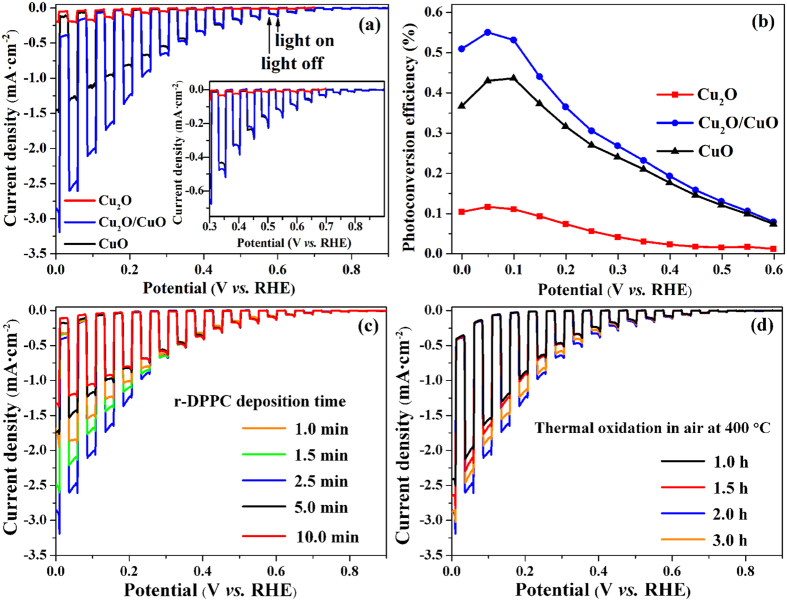
(**a**) Current density *vs.* cathodic potential curves of the Cu_2_O (read line), CuO (black line), and Cu_2_O/CuO bilayered composite (blue line) photocathodes. (**b**) Applied bias photo-to-current efficiency as a function of applied cathodic potential for the Cu_2_O (read line), CuO (black line), and Cu_2_O/CuO composite (blue line) photocathodes. (**c**) Effect of Cu_2_O electrodeposition time on the PEC activity of the Cu_2_O/CuO bilayered composites. (**d**) Effect of the thermal oxidation time on the PEC activity of the Cu_2_O/CuO bilayered composites. All the current-potential curves were obtained in 0.5 M Na_2_SO_4_ solution at pH 6.0 under chopped illumination (100 mW·cm^−2^).

Figure 5a shows the current density-potential responses of the Cu_2_O, the CuO, and the Cu_2_O/CuO bilayered composite photocathodes in 0.5 M Na_2_SO_4_ solution at pH 6.0 under chopped AM 1.5 light illumination. All photocathodes exhibit a reductive photocurrent, which was mainly ascribed to the PEC water reduction^28^,^29^,^30^,^31^,^32^,^33^,^34^,^35^,^36^,^37^,^38^,^39^,^41^,^42^,^43^,^44^,^45^,^46^,^47^,^48^,^49^,^50^,^51^,^52^. The onset potential of the photocurrent of the Cu_2_O film is approximately 0.45 V *vs.* RHE, which is in agreement with previous reports^38^,^50^. The photocurrent density of the Cu_2_O film is not high, with a value of ca. −0.21 mA cm^−2^ at 0.0 V *vs.* RHE. However, after thermal oxidation at 400 °C for 2 h, the resulting Cu_2_O/CuO bilayered composite film exhibits greatly enhanced photoactivity compared to pure Cu_2_O film. As can be seen in the inset of Fig. 5a, the onset potential of photocurrent positively shifts from ca. 0.45 V to ca. 0.80 V *vs.* RHE. The photocurrent density at 0 V *vs.* RHE exceeds −2.47 mA·cm^−2^, which is over 10 and 2 times higher than those obtained on Cu_2_O (red line) and CuO (black line) films, respectively. Moreover, it should be pointed out that this value is not only larger than all the values reported recently on pure copper oxide photocathodes^30^,^39^,^40^,^41^,^47^,^48^,^49^,^50^,^51^, but also larger than some of those obtained on the co-catalyst-modified copper oxides^44^,^45^,^46^,^50^,^52^. However, it should also be pointed out here that, in neutral solution, some of the copper oxide photocathodes that were modified with ZnO, TiO_2_, and electrochemical co-catalyst exhibited higher activity than the Cu_2_O/CuO bilayered composite prepared in this work^28^,^40^,^41^,^42^,^43^.

The applied bias photon-to-current efficiency (*η*), which is defined by the following equation (1), can be used to estimate the PEC performance of photoelectrode in the presence of an external applied potential^10^,^73^,^74^.


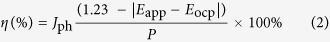


where *J*_ph_ is the photocurrent density (mA·cm^−2^), *E*_app_ is the applied potential at which *J*_ph_ was measured, *E*_ocp_ is the open circuit potential in the same solution and under the same illumination of light at which *J*_ph_ was measured, *P* is the incident light power density (mW·cm^−2^). In 0.5 M Na_2_SO_4_ solution under AM 1.5 light illumination, the values of *E*_ocp_ for Cu_2_O, CuO, and Cu_2_O/CuO composite are 0.68 V, 1.10 V, and 1.05 V *vs*. RHE, respectively. Figure 5b presents the variation of *η* as a function of *E*_app_ for the Cu_2_O, CuO, and Cu_2_O/CuO composite photocathodes. The Cu_2_O and CuO exhibit an optimal conversion efficiency of 0.12% and 0.43% at 0.05 V and 0.10 V *vs*. RHE, respectively. While the Cu_2_O/CuO composite photoelectrode achieved a much higher optimal conversion efficiency of 0.55% at 0.05 V vs RHE, implying that the Cu_2_O/CuO bilayered composite film utilizes the light more efficiently compared to the Cu_2_O and CuO films.

The total thickness and the Cu_2_O-to-CuO thickness ratio of the composite exerted a great effect on the photoactivity by influencing both the light absorbing property and the resistance of the composite film. To optimize the photoactivity, the PEC performance of the Cu_2_O/CuO bilayered composite was investigated as a function of both the electrodeposition time and the thermal oxidation time, because the former determined the total film thickness (see Figure S3 of supplementary material) and the latter controlled the Cu_2_O-to-CuO thickness ratio (see Figure S4 of supplementary material). Figure 5c clearly demonstrates that the photocurrent density first increases and then decreases with increasing deposition time, and the best PEC performance was obtained with a deposition time of 2.5 min. We believe that this observation is due to the following two opposite effects. The light absorption increases with increasing film thickness, resulting in an enhancement of the photocurrent density. However, thicker Cu_2_O/CuO film results in larger ohmic resistance and more grain boundaries, which works against the improvement of the photocurrent density. Hence, the maximum photocurrent density is the result of the balance between the two opposite effects. Figure 5d demonstrates that the thermal oxidation time also has a significant impact on the PEC activity, and the maximum photocurrent density is obtained with a thermal oxidation time of 2 h, which corresponds to a Cu_2_O-to-CuO thickness ratio of ca. 1:1 (see Figure S4 of supplementary material). According to the above discussion, the Cu_2_O/CuO bilayered composite prepared with an electrodeposition time of 2.5 min and a thermal oxidation time of 2 h exhibits the optimal PEC activity for HER.

**Figure 6:**
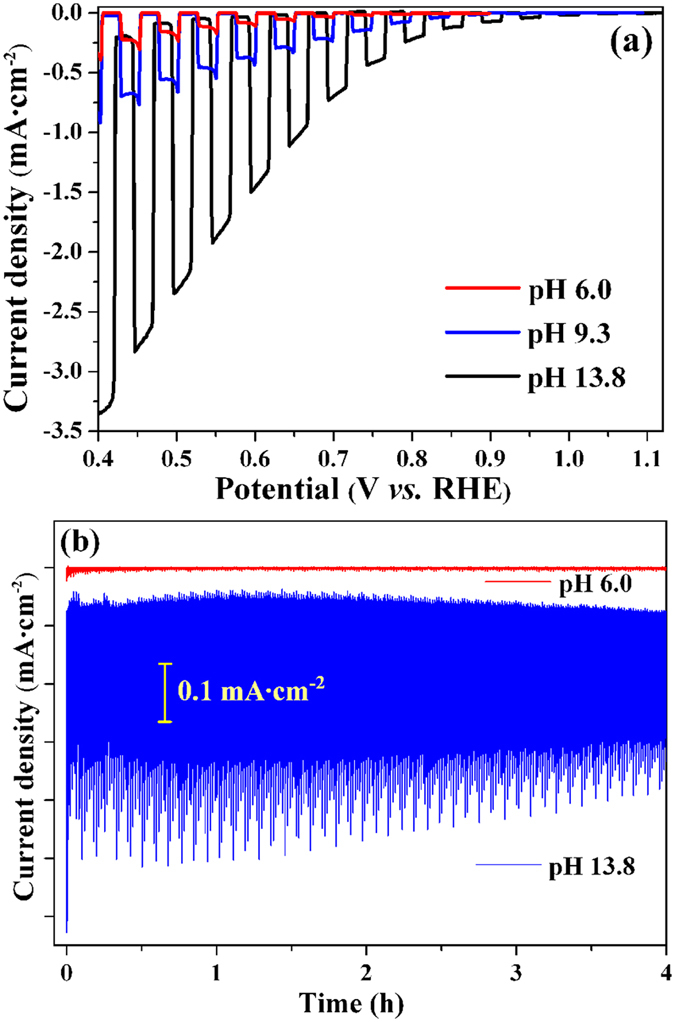
Activity (**a**) and stability (**b**) of the Cu_2_O/CuO bilayered composites in 0.5 M Na_2_SO_4_ solution with different pH values. The Cu_2_O/CuO composites were prepared by r-DPPC deposition for 2.5 min followed by thermal oxidation at 400 °C for 2h. Stability measurements were performed at a relative high HER potential of 0.76 V vs. RHE.

Figure 6a shows the PEC activity of the Cu_2_O/CuO bilayered composite in 0.5 M Na_2_SO_4_ solution with different pH values. The composite photocathode exhibits surprisingly high PEC activity toward HER in an alkaline solution. The onset potential of the photocurrent shifts positively from ca. 0.80 V *vs*. RHE in solution with pH 6.0 (red line) to ca. 1.0 V *vs*. RHE in solution with pH 13.8 (black line). As all potentials are reported with respect to RHE, the thermodynamic effect of the pH value of solution is ruled out. Therefore, the positive onset potential of 1.0 V *vs*. RHE suggests that the Cu_2_O/CuO bilayered composite is highly active for PEC HER in an alkaline solution. In fact, the bilayered composite has a surprisingly high photocurrent density at high potential region (>0.4 V *vs*. RHE). Figure 6a clearly shows that the photocurrent density reaches 1.50 mA·cm^−2^ and 3.15 mA·cm^−2^ at potentials of 0.60 V and 0.40 V *vs*. RHE, respectively. These values are over three times larger than the values obtained on the same electrode at pH 6.0 and pH 9.3. More importantly, these values are among the two highest^34^ obtained in the same potential region at all reported copper-oxide-based photocathodes, including those modified with wide-bandgap semiconductors and electrochemical co-catalysts^28^,^29^,^30^,^31^,^32^,^33^,^34^,^35^,^36^,^37^,^38^,^39^,^40^,^41^,^42^,^43^,^44^,^45^,^46^,^47^,^48^,^49^,^50^,^51^,^52^. Besides ours, the other reported highest photoactivity at high potential region (>0.4 V *vs*. RHE) was obtained on 3-dimentional (3D) copper oxide photocathodes^34^, whose photocurrent density was 1.80 mA·cm^−2^ and 3.15 mA·cm^−2^ at 0.60 V and 0.42 V *vs*. RHE, respectively. These values were slightly higher than those obtained in this work. However, it should be pointed out that the 3D photocathodes in that work were prepared by a biological-template-assisted method which involved several complex biological and chemical procedures^34^. Compare to the above approach, ours provided an ease, low-cost, and scalable strategy to prepare the copper-oxide-based photocathodes for hydrogen production.

With respect to PEC HER, it is highly desirable for copper oxide photocatalysts to exhibit high activity in a more positive potential region because of the following two reasons. (1) To obtain the same photocurrent density, more positive potential means smaller HER overpotential, and then smaller external electrical energy input. (2) As the copper oxides are ready to be reduced at lower potentials^28^,^31^, the more positive potential ensures a much lowered reductive decomposition rate of copper oxides during PEC HER, which benefits the stability of photocathode. Therefore, the high photoactivity at high positive potentials is very important for cupric and cuprous oxide catalysts. However, CuO and Cu_2_O usually have a very low activity toward PEC HER in high potential regions. The photocurrent densities reported in literature on the CuO and Cu_2_O photocathodes without HER co-catalysts were very small at potentials more positive than 0.40 V *vs*. RHE^28^,^30^,^31^,^36^,^37^,^38^,^39^,^40^,^41^,^42^,^43^,^44^,^45^,^46^,^48^,^49^,^50^,^51^,^52^. Accordingly, the high photocurrent density at high positive potentials (Fig. 6a) suggests that the Cu_2_O/CuO bilayered composite is an ideal candidate as a photocathode material for HER.

The photostability of the Cu_2_O/CuO bilayered composite, which is very important for long-term PEC HER, was investigated in 0.5 M Na_2_SO_4_ solution at a relative high potential of 0.75V vs. RHE under chopped illumination. At such a high applied potential of 0.75V vs. RHE, the Cu_2_O film showed a low activity and photostability with its photocurrent density quickly decaying to a neglectable value (data not shown). However, after the formation of Cu_2_O/CuO heterojunction, both the activity and stability of the photocathode were greatly improved. Figure 6b shows the variation of the photocurrent density of the Cu_2_O/CuO bilayered composite as a function of reaction time during PEC HER at 0.75V vs. RHE. The photocurrent density of the composite in alkaline solution is much larger than that in neutral solution, in agreement with results shown in Fig. 6a. Moreover, the Cu_2_O/CuO bilayered composite exhibited good photostability during the 4 h measurements. After 4 h reaction, the average photocurrent density still remains ca. 85% of the average value of the first half hour, indicating a decay of less than 15%. In fact, this is a rather small decay for a copper oxide photocathode without protecting layer. It can be concluded that the formation of outside CuO layer protects Cu_2_O film from corrosion, thus enhancing the photostability^49^,^50^. We believe that modifying the Cu_2_O/CuO bilayered composite with nanometer-thick protecting layer (such as TiO_2_, ZnO, Al_2_O_3_, and carbon) and cocatalyst can further improve the photostability.

## Discussion

**Figure 7:**
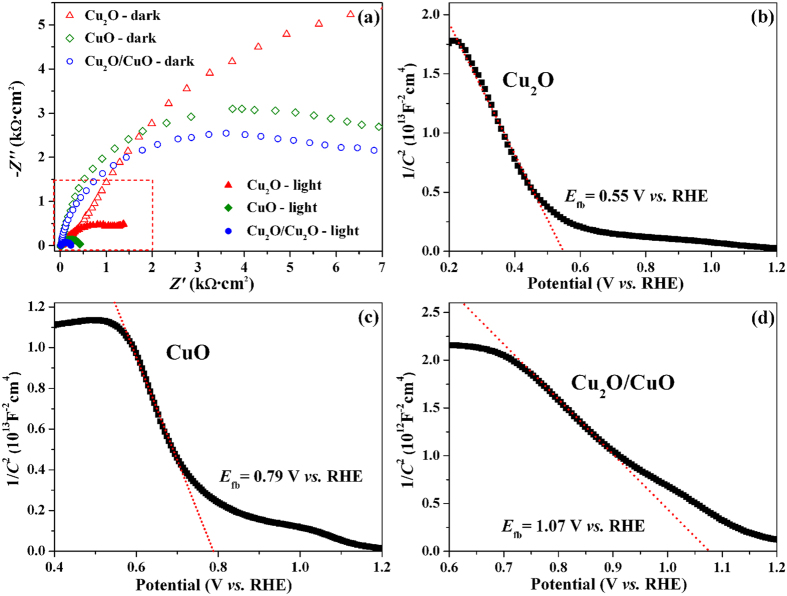
(**a**) Nyquist plots of the Cu_2_O, CuO, and Cu_2_O/CuO bilayered composite at constant potential of 0.3 V vs. RHE in the dark and under illumination. Mott-Schottky plots of (**b**) Cu_2_O, (**c**) CuO, and (**d**) Cu_2_O/CuO bilayered composite.

In order to clarify the origin of the high photoactivity of the Cu_2_O/CuO bilayered composite, electrochemical impedance spectroscopy (EIS) and Mott-Schottky plots^75^,^76^ were employed to investigate the charge transfer rate at the semiconductor/solution interface and the majority carrier density inside copper oxides. Figure 7a shows the Nyquist plots of the three photocathodes at a potential of 0.30 V *vs*. RHE in the dark and under illumination. The semicircle at low frequencies features the charge transfer across the semiconductor/electrolyte interface and the diameter of the semicircle represents the charge transfer resistance (*R*_ct_). As shown in Fig. 7a, *R*_ct_ decreases significantly upon illumination for all photocathodes, indicating that illumination greatly accelerates the charge transfer reaction at copper oxide/solution interface due to the photoinduced increase of carrier density. The variation trend of *R*_ct_ with photocathode composition agrees well with that of photocurrent density, and the Cu_2_O/CuO bilayered composite has the smallest *R*_ct_ value both in the dark and under illumination, suggesting that the Cu_2_O/CuO heterojunction accelerates the charge transfer across the photocathode/solution interface. This explains the reason why the bilayered composite exhibited the best PEC performance for HER.

The space-charge capacitance (*C*_SC_) of semiconductor varies as a function of the applied potential according to Mott-Schottky equation shown below^75^,^76^, which can be used to estimate the flat band potential and the majority carrier density of semiconductor.


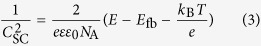


where *N*_A_ is the acceptor density (majority carrier density, i.e. hole density in p-type semiconductors such as CuO and Cu_2_O), *ε*_0_ is the permittivity of the vacuum, *ε* is the dielectric constant of the semiconductor (for CuO and Cu_2_O, *ε* is 10.26 and 7.60, respectively)^36^,^49^,^60^, *E* is the applied potential, *E*_fb_ is the flat band potential, *e* is the electron charge, *k*_B_ is the Boltzmann’s constant, and *T* is the absolute temperature. Figure 7b**–**d show the Mott-Schottky plots of Cu_2_O, CuO, and Cu_2_O/CuO bilayered composite, from which the negative slopes were obtained for three materials, indicating that they were all p-type semiconductors. On the basis of equation (3), the slope of the linear part of the curve in Mott-Schottky plot can be used to estimate *N*_A_, which reflects the majority carrier density. The *N*_A_ values calculated from Fig. 7b–d are 3.07 × 10^17 ^cm^−3^, 2.41 × 10^18 ^cm^−3^, and 2.58 × 10^18 ^cm^−3^ for Cu_2_O, CuO, and Cu_2_O/CuO bilayered composite, respectively. The highest majority carrier density of the Cu_2_O/CuO bilayered composite signifies a fast charge transfer, and thus an enhanced PEC performance^77^. Furthermore, from the intercept of the Mott-Schottky plots (Fig. 7), the flat band potential (*E*_fb_) for Cu_2_O, CuO, and Cu_2_O/CuO composite are estimated to be 0.55 V, 0.79 V, 1.07 V *vs*. RHE, respectively. As is well known, a higher *E*_fb_ value for a p-type semiconductor implies a higher degree of band bending and a larger space-charge-region potential. Therefore, the high *E*_fb_ value of the bilayered composite provides a large driving force for the photo-induced electron-hole pairs to separate in the space charge region, and then results in a high photoactivity toward HER.

To elucidate the advantages of the Cu_2_O/CuO heterojunction during PEC HER, we need to know the locations of band edge of Cu_2_O and CuO. The valence band edge *E*_V_ in eV can be obtained by the following equation:^76^


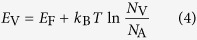


where *E*_F_ is the Fermi level in eV, *N*_V_ is the effective state density in the valence band. *E*_V_ and *E*_F_ can also be expressed in V according to the following equation:


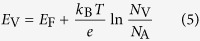


When a semiconductor/solution interface is under intense illumination (for example, AM 1.5 illumination), the energy band bending is minimized and both the conduction and valence bands are flat, as can be seen from the agreement of onset potential of photocurrent with *E*_fb_. Under this flat band condition, *E*_F_ is equal to *E*_fb_. In equations (4) and (5), *N*_V_ can be obtained by:^76^


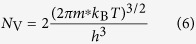


where the effective mass m^*^ of the holes was 0.58*m*_0_ and 7.9*m*_0_ for Cu_2_O and CuO with *m*_0_ as the mass of the free electron^78^,^79^. Hence, *N*_V_ calculated for Cu_2_O and CuO were 1.11 × 10^19 ^cm^−3^ and 5.57 × 10^20 ^cm^−3^. On the basis of equation (5), the valence band edge *E*_V_ of Cu_2_O and CuO are calculated to be at ca. 0.64 V and 0.88 V vs. RHE. As the band gaps of Cu_2_O and CuO are 2.47 eV and 1.58 eV (Fig. 4d), respectively, the conduction band edges of the two oxides can be calculated, which are ca. −1.83 V and −0.70 V vs. RHE for Cu_2_O and CuO, respectively.

**Figure 8:**
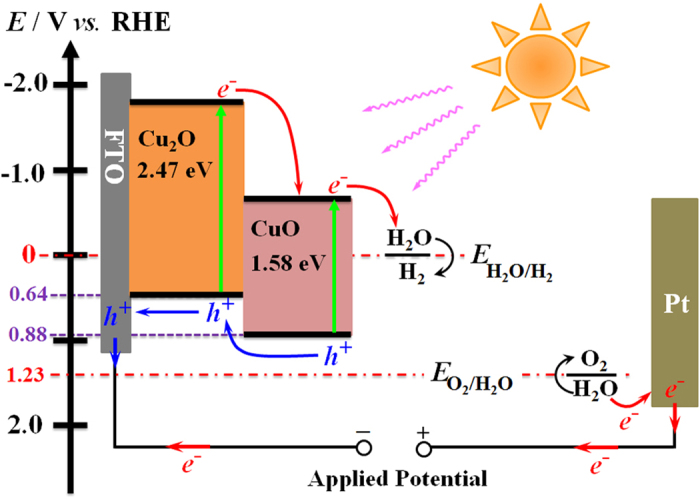
The schematic representation of the energy band diagram of the Cu_2_O/CuO bilayered composite in contact with solution during PEC HER.

Figure 8 shows the band energy structure of Cu_2_O/CuO bilayered composite in 0.5 M Na_2_SO_4_ with pH 6.0 under illumination. The conduction band edges of Cu_2_O and CuO are both more negative than the redox potential of H_2_O/H_2_, indicating that the photo-induced electrons in the conduction bands can be injected into water and result in the reduction of water. The greatly enhanced photoactivity of the Cu_2_O/CuO bilayered composite for PEC HER was attributed to the following three reasons. Firstly, the CuO layer grew on the surface of Cu_2_O markedly broadens the solar absorption region due to its narrower band gap, resulting in a much more efficient utilization of light. Secondly, the formation of Cu_2_O/CuO heterojunction ensures a large space-charge-region potential, which significantly improves the electron-hole separation efficiency and lowers the electron-hole recombination rate. In other words, the large space-charge-region potential at Cu_2_O/CuO interface facilitates not only the injection of photoinduced electrons from the conduction band of Cu_2_O to that of CuO but also the injection of photoinduced holes from the valence band of CuO to that of Cu_2_O. Finally, the Cu_2_O/CuO bilayered composite has a greatly improved charge carrier density, and thus ensures a faster carrier transportation rate inside the copper oxides, which benefits the PEC HER.

In conclusions, the Cu_2_O/CuO bilayered composite photocathodes were successfully prepared by a two-step method that involved an electrodeposition in which the Cu_2_O film was grown on FTO substrate and a subsequent thermal oxidation in which the outer layer of the film was transformed to CuO. The total thickness of the composite film and the relative thickness of the Cu_2_O and CuO layers could be easily controlled by varying the electrodeposition and thermal oxidation time. The light absorption edge of the photocathode was red shifted from ca. 600 nm to ca. 900 nm after the formation of outer CuO layer, extending the utilization of solar energy. The Cu_2_O/CuO bilayered composite showed high activity and good stability for PEC HER, especially at high positive region in alkaline solution. The photocurrent density obtained on the bilayered composite was 3.15 mA·cm^−2^ at 0.40 V *vs*. RHE in 1.0 M KOH solution. This value is one of the two highest reported on copper-oxide-based photocathode at the same potential under 100 mW·cm^−2^ illumination. Moreover, the Mott-Schottky measurements demonstrated that the bilayer composites had a large carrier density (2.58 × 10^18 ^cm^−3^) and a high flab band potential (1.07 V *vs*. RHE), implying the composite has a good conductivity and a high degree of band bending. We believe that the extended light absorption region, the large space-charge-region potential, and the high carrier density of the composite are three main reasons that are responsible for its high photoactivity toward HER. This work demonstrates that the Cu_2_O/CuO bilayered composite is a promising candidate as a photocathodic material for efficient PEC HER. Moreover, the strategy of combining electrodeposition and thermal oxidation provides an ease, low-cost, and scalable approach to fabricating copper-oxide-based materials for hydrogen production.

## Methods

### Chemicals and Materials

Copper sulfate pentahydrate (CuSO_4_·5H_2_O) and lactic acid were purchased from Sinopharm Chemical Reagent Co., Ltd. Sodium hydroxide (NaOH) and anhydrous sodium sulfate (Na_2_SO_4_) were purchased from Beijing Chemical Reagents Company. All chemicals were of analytical reagent grade and used without further purification. The fluorine-doped tin oxide (FTO) glass (8 Ω·sq^−1^, transparency 80%, Asahi Glass, Japan) was used as the conductive substrate. All aqueous solutions were prepared with deionized water (resistance>18 MΩ·cm).

### Preparation of Cu_2_O, CuO, and Cu_2_O/CuO bilayered composite photocathodes

The Cu_2_O films were prepared by a repeated double-potential pulse chronoamperometric (r-DPPC) deposition method. In brief, the electrodeposition of Cu_2_O was performed on a CHI660C electrochemical workstation (CH Instruments Co.) using a three-electrode system, in which FTO substrates served as the working electrodes, and a platinum sheet and a saturated calomel electrode (SCE) as the counter and reference electrodes, respectively. One cycle double-potential pulse chronoamperometric (DPPC) deposition was conducted at −0.5 V *vs.* SCE for 2 s and at 0.0 V *vs.* SCE for 4 s, respectively, in a solution containing 0.48 M CuSO_4_ and 3 M lactic acid, which was adjusted to pH 9.2 with 5 M NaOH. The thickness of the Cu_2_O films was controlled by varying the total deposition cycles (or deposition time). In detail, one deposition cycle costed 6 s, which included 2 s at −0.5 V *vs.* SCE and 4 s at 0.0 V *vs.* SCE. Therefore, taking 6 s as a time unit, we could control the thickness of the Cu_2_O films by controlling the total deposition cycles, especially when we knew that the growth rate of DPPC deposition was *ca*. 10 nm per cycle (corresponding to 100 nm·min^−1^, see the Results section). The Cu_2_O/CuO composite and the pure CuO photocathodes were prepared by thermal oxidation of the Cu_2_O film in air at 400 °C for 2 and 4 h, respectively.

### Characterization

The morphology of the prepared copper oxide photoelectrodes was characterized by field emission scanning electron microscopy (FE-SEM) (Hitachi S-4800, Japan). The crystalline structure was examined by X-ray diffraction (XRD) (Rigaku, Rint 2000 advance theta-2theta powder diffractometer) with Cu Kα radiation. High-resolution transmission electron microscopic (HRTEM) and selected-area electron diffraction (SAED) measurements were performed on a field emission JEM-2010F microscope (JEOL Ltd., Japan) with an accelerating voltage of 200 kV. The UV-Vis diffuse reflectance spectra were collected on a double beam UV-Vis spectrophotometer (Purkinje General, China).

### Electrochemical and photoelectrochemical measurements

All electrochemical and photoelectrochemical (PEC) measurements were performed on a CHI660C electrochemical workstation in a three-electrode cell with a Pt sheet and an SCE as counter and reference electrodes, respectively. The photoresponse was measured under a continuous or chopped irradiation from a 300 W Xe lamp, and the intensity of the light source was calibrated with a FZ-A irradiatometer (Photoelectric Instrument Factory of Beijing Normal University) to simulate AM 1.5 illumination (100 mW·cm^−2^). The area of all photocathodes exposed to light was 0.25 cm^2^. The effect of pH value of the solution on the PEC activity and stability were investigated in nitrogen-purged solutions of 0.5 M Na_2_SO_4_, 0.1 M sodium tetraborate, and 1 M KOH, whose pH values were (or adjusted to) 6.0, 9.3 and 13.8, respectively. Before PEC measurements, the solution was purged with N_2_ for 30 minutes to remove O_2_. Unless indicated, all the potentials were calibrated to reversible hydrogen electrode (RHE) according to the following equation:


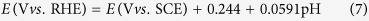


The Mott-Schottky plots were obtained at a frequency of 1 KHz and an amplitude of 10 mV to determine the flat-band potential. Electrochemical impedance spectroscopic measurements were carried out in the dark and under illumination at an AC voltage of 10 mV with a frequency region ranging from 0.1 Hz to 100 kHz.

## Additional Information

**How to cite this article**: Yang, Y. *et al.* Cu_2_O/CuO Bilayered Composite as a High-Efficiency Photocathode for Photoelectrochemical Hydrogen Evolution Reaction. *Sci. Rep.* **6**, 35158; doi: 10.1038/srep35158 (2016).

## Supplementary Material

Supplementary Information
