# Supplementary material for: Cu2O/CuO Bilayered Composite as a High-Efficiency Photocathode for Photoelectrochemical Hydrogen Evolution Reaction
Source: Sci Rep. 2016 Oct 17;6:35158. doi: 10.1038/srep35158 (PMC5066255; doi:10.1038/srep35158)
Supplement: Supplementary Information [file srep35158-s1.pdf]

Supplementary Information

Cu_2O/CuO Bilayered Composite as a High-Efficiency Photocathode for Photoelectrochemical Hydrogen Evolution Reaction

Yang Yang, Di Xu, Qingyong Wu, Peng Diao*

Key Laboratory of Aerospace Materials and Performance (Ministry of Education), School of Materials Science and Engineering, Beihang University, Beijing 100191, P. R. China

* Corresponding author. Tel. & Fax: 86-01-82339562
E-mail address: pdiao@buaa.edu.cn (P. Diao)

Figure S1

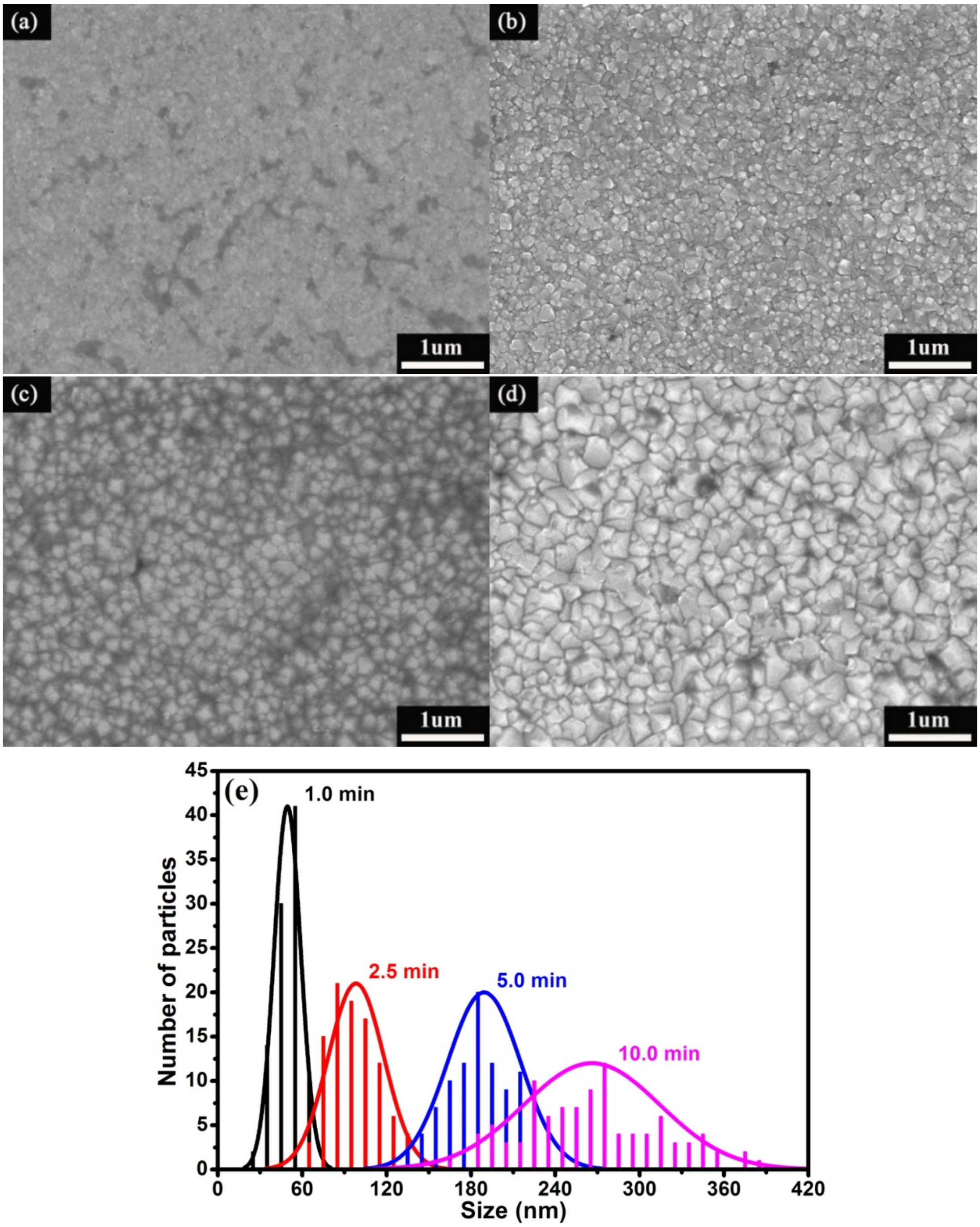

Figure S1. Top-view SEM images of Cu_2O films prepared on FTO substrates by the repeated double-potential pulse chronoamperometric (r-DPPC) deposition method. The r-DPPC deposition was carried out by repeating the two potential pulses at -0.5 V *vs.* SCE for 2 s and at 0.0 V *vs.* SCE for 4 s. The total deposition time: (a) 1.0 min, (b) 2.5 min, (c) 5.0 min, (d) 10.0 min. (e) The corresponding grain size distributions of the Cu_2O films prepared with different deposition time. The deposition solution contains 0.48 M $CuSO_4$ and 3 M lactic acid, which was adjusted to pH 9.2 with 5 M NaOH.

Figure S2

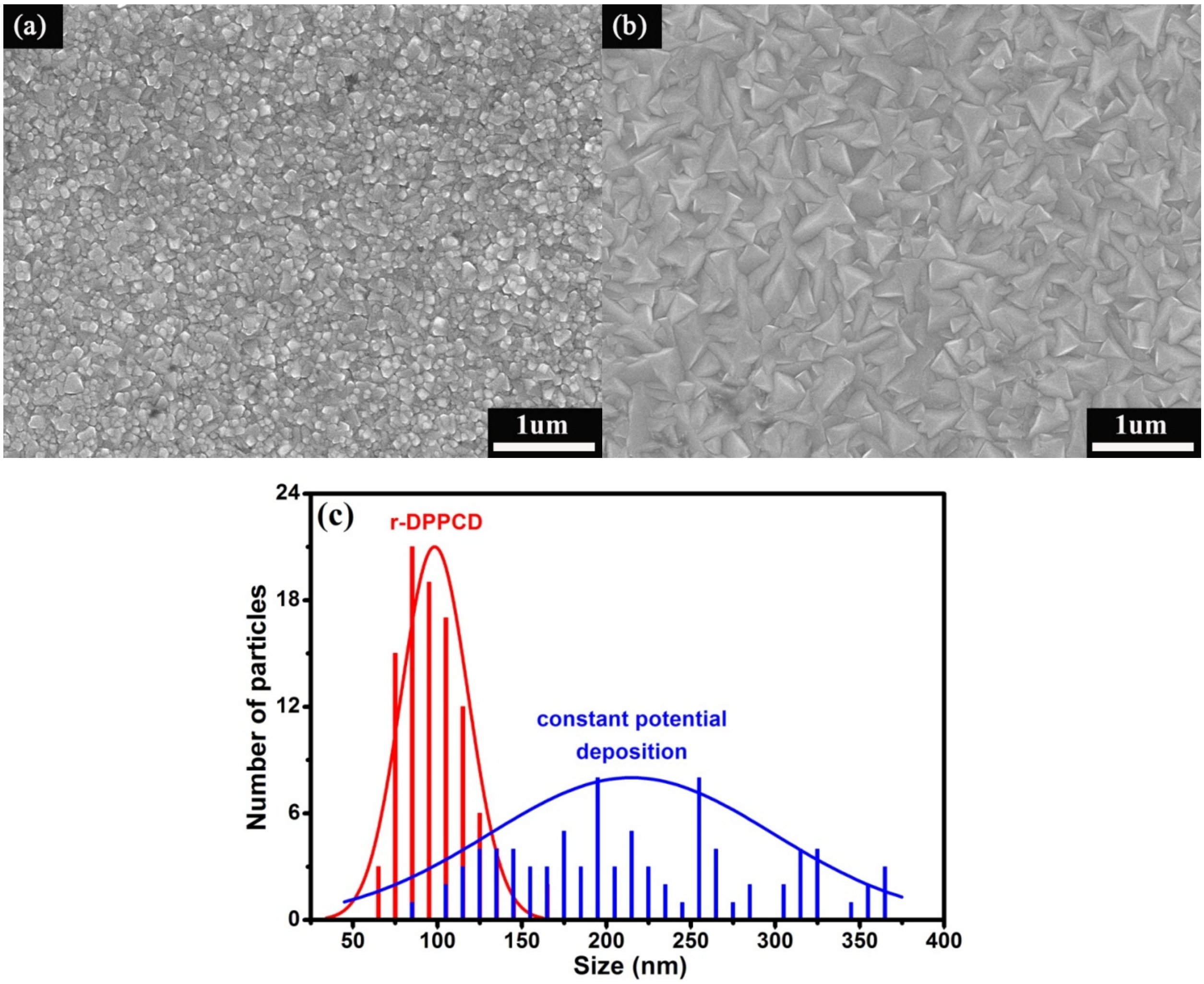

Figure S2. Top-view SEM images of Cu_2O film prepared by (a) r-DPPCD and (b) constant potential deposition. The r-DPPCD was carried out by repeating the two potential pulses (at -0.5 V *vs.* SCE for 2 s and at 0.0 V *vs.* SCE for 4 s) for 2.5 min (25 DPPCD cycles). The constant potential deposition was carried out at -0.5 V *vs.* SCE for 2.5 min. For both methods, the electrodeposition was performed in a solution containing 0.48 M $CuSO_4$ and 3 M lactic acid, which was adjusted to pH 9.2 with 5 M NaOH.

Figure S3

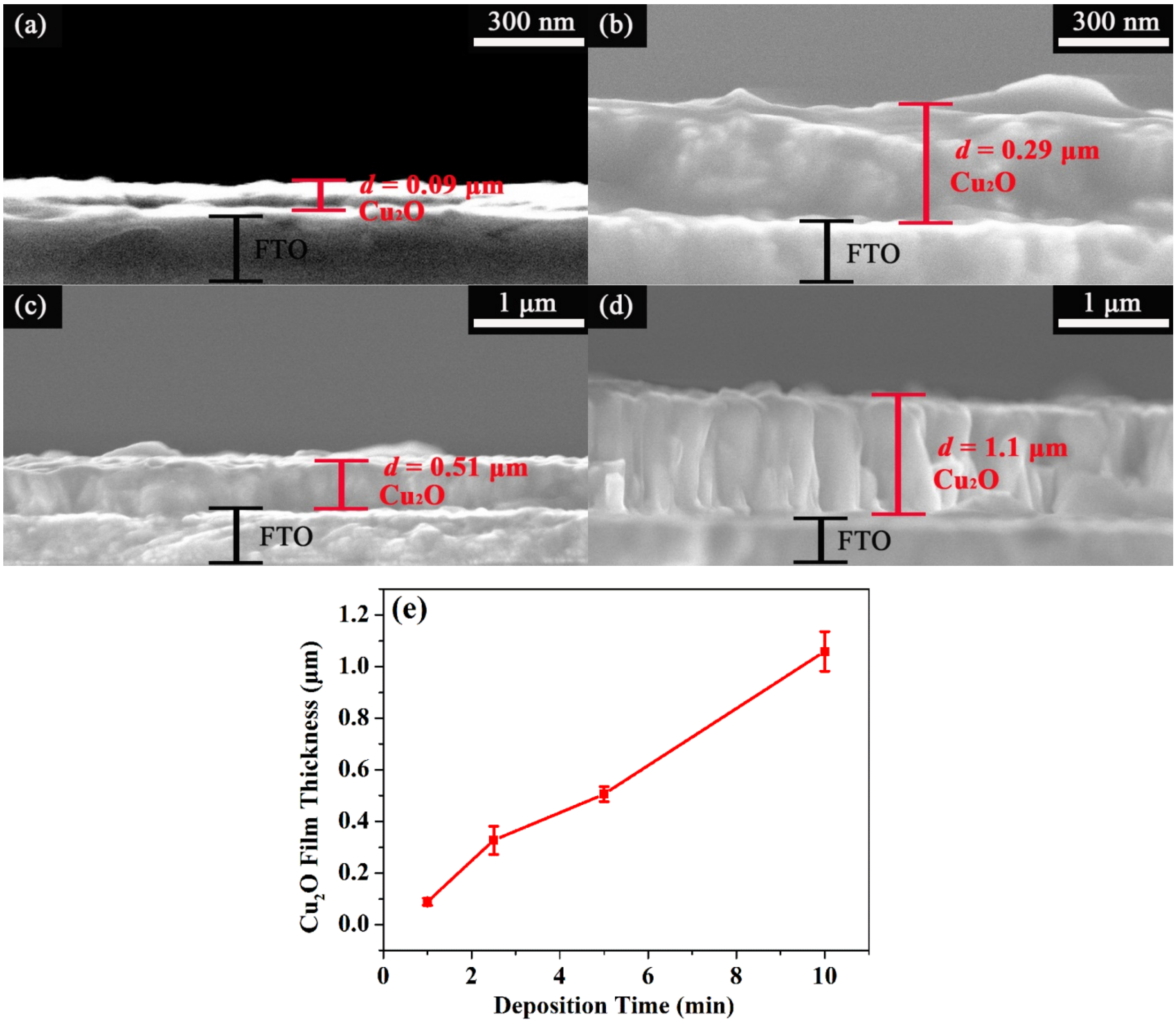

Figure S3. The cross-sectional SEM images of the Cu_2O films prepared on FTO substrates by r-DPPC deposition with different deposition time: (a) 1.0 min (b) 2.5 min, (c) 5.0 min, (d) 10.0 min. (e) Variation of the thickness of Cu_2O film as a function of deposition time.

Figure S4

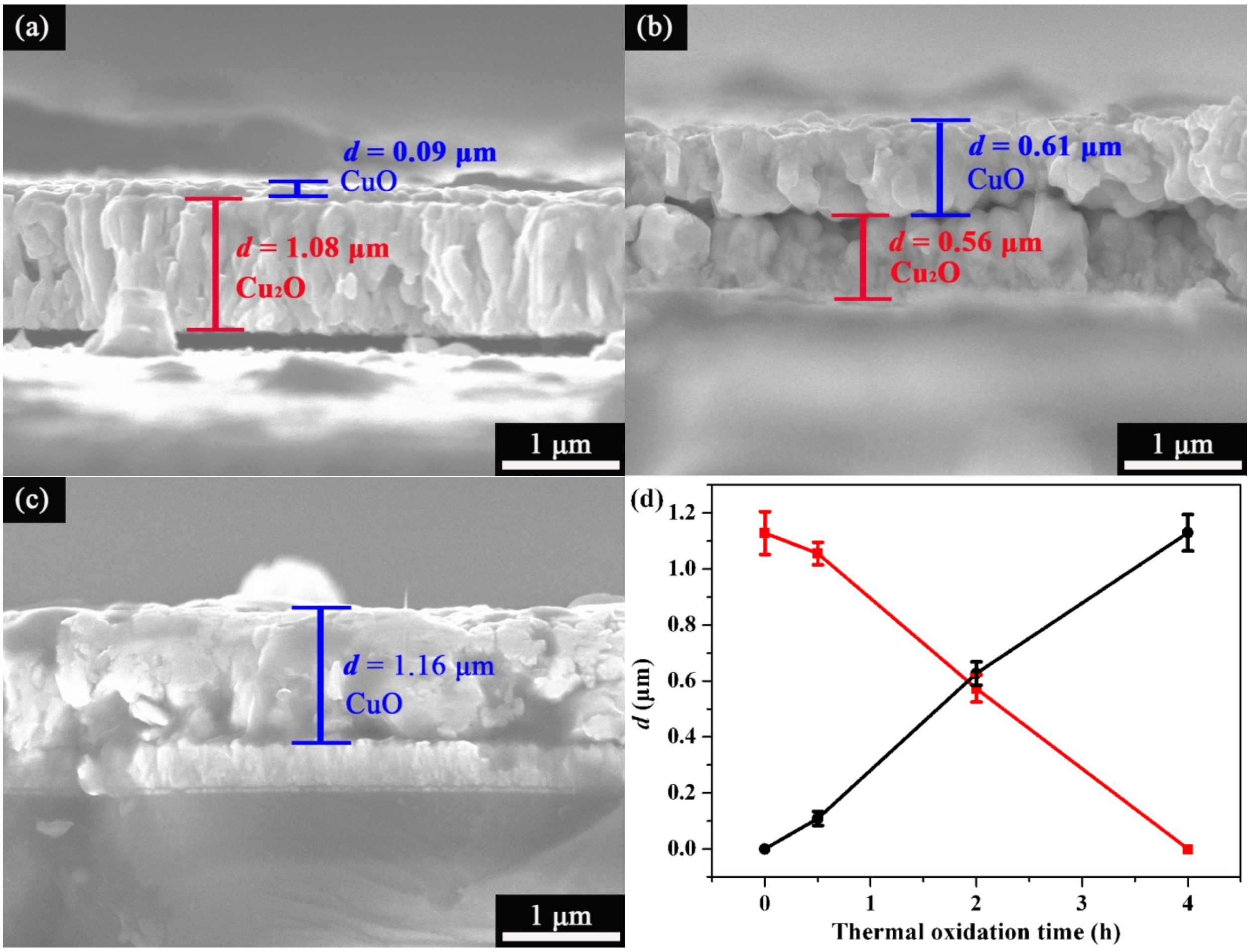

Figure S4. The cross-sectional SEM images of Cu_2O/CuO bilayered composite films prepared by thermal oxidation of the electrodeposited Cu_2O film at 400°C for (a) 0.5 h, (b) 2.0 h, (c) 4.0 h. (d) Variation of the thicknesses of the Cu_2O layer (red) and the CuO layer (black) as a function of thermal oxidation time. The Cu_2O films, which were used to prepare the Cu_2O/CuO bilayered composite, were prepared by r-DPPC deposition for 10 min and had a film thickness of ca. 1.1 μm.
